# Effects of Alcohol and HIV Infection on the Central Nervous System

**Published:** 2001

**Authors:** Dieter J. Meyerhoff

**Affiliations:** Dieter J. Meyerhoff, Ph.D., is an associate professor in the Department of Radiology and Department of Veterans’ Affairs Medical Center, University of California San Francisco, San Francisco, California

**Keywords:** HIV infection, central nervous system, immune system, heavy AOD (alcohol and other drug) use, chronic AODE (effects of AOD use, abuse, and dependence), drug therapy, patient compliance, brain damage, neuropathy

## Abstract

Many people at risk for or infected with the human immunodeficiency virus (HIV) are heavy drinkers. Both HIV infection and heavy alcohol use adversely affect the immune system and central nervous system (CNS) function. However, little research has addressed the effects of heavy alcohol use on the severity and progression of HIV disease, including the development of HIV-associated CNS disease. Animal and in-vitro studies suggest that alcohol impairs various aspects of the immune system and increases the susceptibility to HIV infection, but may not accelerate progression of HIV disease. However, heavy alcohol use may interfere with the patient’s adherence to antiretroviral treatment regimens. Neuropathological and neuropsychological studies have indicated that certain brain areas are affected by both HIV-infection and chronic alcohol abuse. Magnetic resonance spectroscopy studies of both HIV-positive and HIV-negative people who were either heavy or light drinkers found that chronic alcohol abuse exacerbates some metabolic injury in the brains of HIV-infected people, although this effect may be less pronounced in patients receiving effective antiretroviral therapy.

Infection with the human immunodeficiency virus (HIV) and the resulting acquired immunodeficiency syndrome (AIDS) epidemic are major public health problems in the United States and worldwide. One common cause of illness and death associated with HIV infection is central nervous system (CNS) disease. The most severe manifestation of CNS disease is HIV-associated dementia complex (HADC), a neurological syndrome characterized by disordered mental functions (i.e., cognition), motor functions, and behavior. Many members of populations at high risk for HIV infection (e.g., intravenous drug users and homosexuals) also are heavy drinkers, and alcohol has its own adverse effects on CNS function. Nevertheless, almost no research has directly addressed the effects of heavy alcohol use on the severity of HIV disease and its progression to AIDS.

This review summarizes information regarding the prevalence of alcohol abuse among HIV-infected people as well as current knowledge regarding the separate and combined effects of HIV infection and alcohol use on the brain and the immune system. The article then reviews the relationship among alcohol use, adherence to HIV treatment regimens, and HIV disease progression. Subsequently, it discusses the CNS effects of HIV infection/AIDS and alcohol abuse, focusing on pathological and psychological observations as well as on findings obtained using various imaging techniques. Finally, the article reviews the scant published literature on the CNS effects of combined HIV infection and alcohol abuse.

## Prevalence of Chronic Alcohol Use Among the HIV-Infected Population

By the end of the year 2000, approximately 920,000 Americans were living with HIV infection/AIDS,^1^ and approximately one-half of these patients had been infected with HIV for more than 10 years. Most HIV-infected people in North America are between ages 18 to 44. Within the general American population of this age range, 17 percent of men and 10 percent of women are afflicted at some point over their lifespan by an alcohol use disorder (AUD)—that is, alcohol abuse or dependence. Among alcohol-abusing patients, the HIV infection rate is significantly higher than for the general population—that is, about 5 to 10 percent (depending on risk factors such as intravenous drug use, homosexuality, and geographic location)*.* (For a more extensive review of alcohol use in HIV-infected patients see, for example, Petry 1999.)

Similarly, AUDs are much more common among HIV-infected people than among the general U.S. population. For example, 29 to 60 percent of HIV-infected patients develop an AUD at some point during their lives, a rate that is approximately three times as high as that of the general U.S. population. In fact, this prevalence rate is one of the highest rates of drug abuse found among any population group in the United States. Moreover, the prevalence of a *current* AUD among HIV-infected patients is nearly 12 percent, about twice the rate of the general population (Petry 1999).

Some researchers have questioned these statistics because of a purported sampling bias. Most of this epidemiological work was conducted during the early phase of the HIV/AIDS epidemic in the mid 1980s and included almost entirely samples of gay men who were not representative of the HIV-infected community as a whole (e.g., the participants were recruited in gay bars) (see Paul et al. 1991). Therefore, the actual AUD prevalence rates may be lower than these early estimates. Nevertheless, a large proportion of HIV-infected people likely is abusing alcohol or is “alcoholic.”^2^ Between 1984 and 1992 (the latest time period for which such data are available), however, the abuse rates for both alcohol and other drugs (with the exception of amphetamines) declined among HIV-infected people, either as a result of aging among HIV-infected men or as a reaction to the ongoing AIDS epidemic (Crosby et al. 1998).

Research into the added risk associated with the presence of an AUD on HIV infection rates and on disease progression to AIDS and HADC is only in its infancy. AUDs may affect HIV-associated CNS disease in two ways. First, heavy chronic drinking may hasten the progression of the overall HIV disease process through biological (e.g., immunological) effects. Second, AUDs may interfere with treatment seeking, treatment adherence, and treatment effectiveness among HIV-infected people. The following sections briefly summarize these effects separately for HIV/AIDS and AUDs as well as for the co-occurrence of both diseases.

## Biological and Immunological Effects of HIV Infection and Chronic Alcohol Use

### Targets of HIV Infection in the Immune System and CNS

The primary target of HIV in the body is a type of immune cell called the CD4+, or T-helper cell,^3^ which plays a pivotal role in the body’s defense against disease-causing microorganisms (i.e., pathogens). HIV-infected CD4+ cells produce new virus and eventually die. As a result, the infected person’s levels of CD4+ cells in the blood slowly decline, leaving him or her increasingly vulnerable to infections by various pathogens that normally are controlled easily by the immune system (i.e., opportunistic infections) and to unusual cancers.

CD4+ cells are not found in the brain, however, and HIV therefore must infect other cells there to cause CNS disease. The primary cell types in the CNS are nerve cells (i.e., neurons) and glial cells, which include the following three major subtypes:

- Astrocytes, which provide structural and functional support for neurons and which are important for the regulation of ion concentrations
- Oligodendrocytes, which extend projections that form a protective sheath (i.e., myelin sheath) around the extensions (i.e., axons) of neurons
- Microglia, which can change into a type of immune cell called phagocyte or macrophage that seeks out and destroys foreign microorganisms and viruses.

HIV does not infect neurons, but it does infect microglia and, to a lesser extent, astrocytes. Once HIV has entered the brain, these “activated” microglia and multiplying astrocytes produce increasing amounts of substances that when present at excessive levels are toxic to neurons (i.e., neurotoxins). The production of neurotoxins is stimulated by compounds called cytokines that act as chemical messengers among immune cells as well as between immune and nonimmune cells. The same cytokines also further enhance the multiplication of astrocytes and microglia (i.e., gliosis) as well as the production of new HIV particles.

Neurotoxins can damage or even kill neurons, and this process is thought to contribute to the neuropsychological and cognitive deterioration that occurs during the natural course of HIV disease. The most serious consequence of this deterioration is HADC, which is found in approximately 15 percent of HIV-infected people, mostly in those patients whose immune systems have already been severely damaged by HIV (i.e., patients who are severely immunosuppressed^4^).

### Effects of Alcohol on the Immune System

Chronic heavy alcohol use is associated with increased illness and even death related to infectious diseases, although alcohol’s specific effect on the immune system in these relationships is controversial. Limited studies conducted in intact animals (i.e., in vivo) and in cultured tissues and cells (i.e., in vitro) suggest that alcohol can interfere with the normal functions of various components of the immune system (e.g., natural killer cells and T-cells), thereby impairing the body’s response to pathogens (for a review, see Szabo 1999). However, little is known about the effects that acute and chronic alcohol consumption have on the function of the peripheral (i.e., not pertaining to the CNS) immune system in humans, and no information exists about alcohol’s effects on the immune function of the CNS (i.e., on the microglia). Animal and human studies suggest that chronic alcohol consumption adversely affects the peripheral immune system, probably by inducing the production of cytokines. If a similar mechanism exists in the CNS, alcohol-induced cytokine release could increase the amount and activity of neurotoxins and, subsequently, lead to the death of neurons. This hypothesis of alcohol-induced neuron damage is consistent with the findings of neuropathological and imaging studies of chronic alcohol abusers.

### Effects of Combined HIV Infection and Alcohol Use on the Immune System

The previous two sections suggest that both HIV infection and chronic heavy drinking have similar and profound effects on the immune system (for extensive reviews, including immunological and behavioral concepts, see Dingle and Oei 1997; Tyor and Middaugh 1999; Petry 1999). Therefore, researchers have postulated that chronic alcohol use by HIV-positive people may exacerbate the immunological abnormalities that are associated with HIV infection. Researchers have begun to analyze the effects of alcohol in HIV-infected subjects using rodent models of HIV infection, in which mice are infected with a virus that is similar to HIV and can develop a condition comparable to AIDS. These studies suggest that both acute and chronic alcohol consumption reduce the animals’ peripheral immune functions, possibly by altering the release of certain cytokines (for reviews, see Tyor and Middaugh 1999; Dingle and Oei 1997). Furthermore, the animals progress more rapidly from the initial viral infection to mouse-type AIDS or death.

Researchers also have conducted studies using immune cells (i.e., lymphocytes)—including CD4+ cells—that were isolated from healthy, non-HIV-infected human subjects both before and after the subjects consumed 3 to 9 alcoholic drinks over 2 days (Bagasra et al. 1993). The studies suggested that alcohol consumption damaged the peripheral immune system, because when the isolated cells were infected with HIV in a test tube, the virus multiplied faster in cells taken after alcohol consumption. Furthermore, cells isolated after alcohol consumption exhibited reduced CD4+ functions compared with cells isolated before alcohol consumption. These findings suggest that alcohol increases the susceptibility of human cells to HIV infection. Alcohol may exert these effects either directly, by acting on CD4+ cells, or indirectly, by altering the production of cytokines. Some of these effects may be reversible, however, because studies on alcoholic HIV-infected patients undergoing alcoholism treatment found that the patients’ levels of CD4+ cells improved after detoxification (Pol et al. 1996).

Although it appears likely that alcohol impairs the activity of the peripheral immune system and increases the susceptibility to HIV infection, the effects of alcohol on human immune function in the CNS and on the susceptibility of brain cells to HIV infection are ambiguous. Similarly, the impact of alcohol use on the progression of HIV disease to AIDS remains unclear. Several immunological studies followed large numbers of alcohol-consuming homosexual men over time but found no evidence that alcohol accelerated disease progression to AIDS (e.g., Kaslow et al. 1989; Penkower et al. 1995). These studies have methodological weaknesses, however. For example, one study followed the participants only over a period of 18 months, which may not have been long enough, considering that it usually takes approximately 3 to 10 years for an HIV-infected person to develop AIDS. Another study only examined the effects of consumption of up to approximately 70 alcohol-containing drinks per month, or a little more than 2 drinks per day, which generally is considered moderate drinking; the study did not assess the consequences of heavier drinking*.* A more recent report suggests, however, that even heavy drinking (i.e., more than 150 drinks per month for an unspecified duration) does not significantly alter the effectiveness of antiretroviral treatment in controlling the virus levels in HIV-infected patients (Fabris et al. 2000). This finding also implies that alcohol consumption does not accelerate the progression of HIV disease.

In summary, animal and in-vitro studies have indicated that chronic alcohol use can have deleterious effects on the immune system. In addition, chronic alcohol use may influence a person’s susceptibility to infections by modifying the nutritional status and promoting the generation of highly reactive molecules that can damage the cells (i.e., oxidative stress) (see Wang and Watson 1995). Thus, it appears plausible that heavy drinking would increase a person’s susceptibility to infection by HIV and accelerate disease progression to AIDS. To date, however, no strong immunological evidence links chronic alcohol consumption at any level to an accelerated course of HIV disease. The paucity of existing immunological data demonstrates a great need for well-controlled, preferably long-term studies to document the combined effects of HIV infection and heavy alcohol consumption on the progression of HIV disease in this era of promising antiretroviral therapy.

### Adherence to HIV Treatment

The currently recommended treatment for patients with HIV infection frequently involves therapy with three different medications from two classes of medications called reverse transcriptase inhibitors and protease inhibitors.^5^ This treatment regimen, which is called highly active antiretroviral therapy (HAART) or potent antiretroviral therapy, can reduce the patient’s risk of opportunistic infections commonly associated with HIV infection and HADC, as well as improve mental abilities and survival rates. The three medications must be administered according to very strict schedules requiring the patient to take more than a dozen tablets at specific times each day, some of them with a meal and others without food. For this treatment to be most effective, the patients must adhere to the schedule approximately 95 percent of the time. Such a regimen requires a level of discipline that many patients cannot maintain. Thus, studies among outpatients indicate that about one-half of HIV-infected patients skip drug doses or pills for a variety of reasons—wanting to avoid side effects associated with the medications, having experienced a change in their daily routines, being busy with other things, being away from home, forgetting, or being depressed. Other factors, such as HIV-associated memory impairment or compromised health, also may reduce a patient’s adherence to the treatment schedule. By not adhering to the complex treatment regimen, patients may not only limit treatment effectiveness but may increase the risk that a treatment-resistant virus develops because the medication levels are too low.

Studies have indicated that HIV-infected patients who use illicit drugs are less willing to begin antiretroviral treatment and have greater difficulty in adhering to a treatment regimen compared with patients who do not use drugs (see Petry 1999). These findings suggest that alcohol-abusing HIV-infected patients also exhibit lower adherence to their treatment regimens than do non-alcohol-abusing patients. Furthermore, because the patients in the existing studies were only taking a single medication, it is reasonable to assume that adherence to the more complex HAART regimen is even lower. Although this issue has not yet been studied, it is likely that nonadherence to antiretroviral treatment is an important factor in the relationship between alcohol abuse and HIV disease progression, including the development of psychiatric symptoms and mental impairment.

An example of this problem can be found among the tuberculosis population. In tuberculosis patients with alcohol or other drug abuse problems, nonadherence to treatment medication has led to the emergence of medication-resistant strains, the frequency of which only decreased with directly observed antituberculosis therapy.

## Effects of HIV Infection and Alcohol Abuse on the CNS

The development of new antiretroviral medications has greatly enhanced treatment effectiveness in controlling the spread of HIV in the body. These medications cannot effectively enter the CNS, however, to eradicate the virus that has infected brain cells. Accordingly, factors that influence CNS disease, such as alcohol abuse, may become even more important in the clinical management of HIV disease and of the HIV epidemic. The separate effects of HIV infection and chronic alcohol abuse on brain structure and function and on biologically relevant molecules that are associated with neuron loss and gliosis are well established. However, almost no research has addressed directly the effect of heavy alcohol use on the severity and progression of HIV-associated CNS disease. This section reviews the main CNS effects of both HIV infection and alcohol abuse by summarizing the pathological and psychological evidence for brain damage associated with both conditions. This discussion also describes structural and some functional correlates of such damage, which can help to understand the cognitive changes occurring in alcohol-abusing HIV-infected patients.

### Neuropathology and Neuropsychology of HIV-Infected People

Neuropathological studies^6^ conducted during the 1990s found that approximately 75 percent of the AIDS patients studied exhibited some kind of CNS damage. Furthermore, a similar proportion of AIDS patients exhibited neurological signs or symptoms before their death, such as memory loss, decreased concentration and attention, and problems with movement or balance (Price 1999). Neuropathological studies also revealed virus-associated abnormalities in various brain areas, including the following (Price et al. 1988; Cummings 1986) (see figure):

- *The basal ganglia.* These are groups of nerve cells located deep inside the brain that help control motor and cognitive activity. The basal ganglia region carries the heaviest HIV load of all brain structures and shows effects of gliosis and inflammation early in the course of the disease. Neuron loss in this region, although it is not a common finding, has been reported in some more recent studies (Masliah et al. 1996).
- *White matter around the fluid-filled spaces (i.e., ventricles) in the brain.* White matter consists primarily of glial cells and axons surrounded by myelin sheaths. In HIV-infected patients, this white matter is infiltrated by various immune cells (i.e., macrophages and lymphocytes). Patients with advanced stages of HIV disease often exhibit abnormal proliferation of glial cells and changes in the myelin sheaths.
- *The cortex.* The cortex is the brain’s outer layer, which is responsible for higher cognitive functions. In advanced stages of AIDS, cortical changes have been reported, including loss of large motor neurons, decreased density of the neuron structures that allow nerve signal transmission among neurons (i.e., synapses), and damaged neuronal extensions (i.e., dendrites) that normally receive incoming nerve signals.

Consistent with these observed abnormalities, HADC patients in neuropsychological studies exhibit a constellation of deficits associated with dementia resulting from damage to brain regions other than the cortex (i.e., subcortical dementia), including deficits in concentration and attention, psychomotor slowing, and mild-to-moderate impairments of memory and abstraction ability (Cummings 1986). Similar impairments are sometimes observed in alcoholic patients. Even though many HIV-infected people are heavy drinkers, a study comparing the neuropsychological functions of HIV-positive and HIV-negative participants suggested that alcohol use did not account for differences in the test scores between the two groups (Bornstein et al. 1993). HAART has been shown to improve neuropsychological test performance of HIV-infected people in some but not all cognitive domains (Tozzi et al. 1999).

### Brain Structure and Metabolites in HIV-Infected People

Researchers have used various imaging techniques, including magnetic resonance imaging (MRI) to analyze changes in the brain structure of HIV-infected people and magnetic resonance spectroscopy (MRS) to measure brain chemicals. For both of these techniques, the patient is exposed to radiowaves in the presence of a powerful magnetic field. This treatment causes the nuclei of hydrogen atoms in the water and chemicals of the different brain tissues to emit signals, depending on their location within the brain and their local surroundings. MR computers translate these signals into two- or three-dimensional images that delineate specific structures (e.g., various brain regions) or into spectra that show characteristic peaks for specific chemicals. MRI and MRS differ in the kind of signal they detect: MRI measures mainly water and, to a lesser extent, fatty substances (i.e., lipids), whereas MRS detects signals from amino acids (which are the building blocks of proteins), building blocks and breakdown products of lipids, and certain molecules involved in energy production. Thus, MRS can detect abnormalities of metabolites in specific brain regions, whereas MRI can detect structural brain abnormalities and can differentiate with great accuracy (i.e., at high spatial resolution) white matter tissue in the brain from gray matter tissue (which consists primarily of neuronal cell bodies and dendrites). MRI also can detect changes in the volumes of both types of tissue. Combined, these two techniques allow a powerful and noninvasive view inside the brain. (For a description of MRS and the molecules it measures, see the sidebar on p. 294).

**Introduction to Magnetic Resonance Spectroscopy**

Like magnetic resonance imaging (MRI), magnetic resonance spectroscopy (MRS) uses radio waves and a strong magnetic field to measure the concentration of naturally occurring metabolic byproducts called metabolites in the human body. This technique causes certain atoms in those metabolites to emit a signal that can be converted into an image, offering researchers a snapshot of the cellular activity taking place within the tissue studied. MRI measures the signals emitted by hydrogen atoms, found primarily in water and fat molecules (i.e., lipids). MRS, in contrast, detects signals emitted by other atoms (i.e., protons and phosphorus atoms), which typically are contained in molecules involved in energy metabolism and other vital cell functions and cell components (e.g., the cell membranes). As a result, MRS is thought to be more specific in measuring tissue damage than MRI. Several metabolites exist that are relevant to HIV and alcohol research and that can be detected by proton and phosphorus MRS of the brain.

Metabolites detectable by proton MRS include the following:

- N*-acetyl-aspartate (NAA).* This protein building block (i.e., amino acid) is found almost exclusively in mature nerve cells (i.e., neurons) and is therefore considered a marker of neuronal viability. In the brain’s gray matter, NAA reflects the viability of neuronal cell bodies whereas in white matter it reflects the viability of the neuronal extensions that transmit nerve signals to other neurons (i.e., the axons). The exact function of NAA in the brain is largely unknown but it is thought to play a role in fat generation and fluid balance.
- *Creatine (Cr) and phosphocreatine (PCr).* These compounds allow the cell to store energy (by converting Cr into PCr) and to release that energy quickly when needed (by converting PCr back into Cr and inorganic phosphate, Pi). Therefore, these molecules reflect cellular energy metabolism in the tissues. PCr and Pi also can be measured using phosphorus MRS.
- *Choline-containing compounds (Cho).* These molecules include several compounds that are building blocks and breakdown products of the membrane surrounding each cell. The primary components of these membranes are phosphorus-containing fat molecules (i.e., phospholipids) Accordingly, Cho reflect various membrane-related metabolic processes, including the generation and breakdown (i.e., turnover) of phospholipids (and, therefore, the membrane) and membrane integrity. In addition, Cho serves as an indicator of inflammation and gliosis—the excessive multiplication of glial cells, which provide a support function to the neurons.
- *Myo-inositol (mIno).* This sugar molecule is believed to be characteristic for glial cells; its concentration is increased in gliosis and with membrane breakdown.

Metabolites detectable by phosphorus MRS include the following:

- *Adenosinetriphosphate (ATP).* This molecule is used for long-term energy storage in the cell and therefore provides a good indicator of energy metabolism.
- *Inorganic phosphate (Pi).* This is a small phosphorus-containing molecule that is crucial for the formation of cellular compounds involved in energy metabolism (i.e., ATP and PCr). Its concentration increases when the cell uses energy.
- *Phosphodiesters (PDE).* This group of molecules consists of several breakdown products of membrane phospholipid components, such as glycerophosphocholine, glycerophosphoethanolamine, and glycerophosphoinositol.
- *Phosphomonoesters (PME).* These molecules include building blocks for phospholipids, such as phosphocholine and phosphoethanolamine. PDE and PME together are measures of membrane turnover and therefore complement Cho measures from proton MRS, providing another indicator of neuron function and integrity.
- *“Broad component” (BC).* This composite signal, which can be measured at a specific static magnetic field strength, results from certain phospholipids within cell membranes. It is possibly a measure of membrane integrity.

Instead of reporting absolute signal intensity or concentrations of the metabolites, researchers often measure the ratios between two of these metabolites for experimental reasons. Commonly used ratios include the NAA/Cr and NAA/Cho ratios. The interpretation of metabolite ratios, however, is sometimes ambiguous, because it may not be clear whether changes in the metabolite in the numerator, denominator, or both cause a change in the ratio. Despite such limitations, however, measurements of the levels of certain metabolites or their ratios can provide researchers with a picture of the health and function of cells within a living organism as well as of the effects that other substances, such as alcohol, have on the function and viability of those cells.

—Dieter J. Meyerhoff

MRI studies of HIV-positive patients who show no symptoms of HIV disease and no dementia (i.e., patients who are asymptomatic) reveal generally mild brain shrinkage (i.e., atrophy) compared with HIV-negative people (for a review see, for example, Navia and Gonzalez 1997). Patients with HADC and/or advanced stages of HIV disease, however, generally exhibit greater brain atrophy affecting many brain areas. Consistent with the neuropathological studies described in the previous section, symptomatic HIV-positive patients in MRI studies showed loss of brain volume particularly in white matter, temporal lobe gray matter, the basal ganglia, and the posterior cortex. In particular, shrinkage of the caudate—a small structure in the basal ganglia related to cognition and motivation that is connected to the prefrontal cortex—appears to be associated with dementia and advanced disease stage.

Although MRI studies can be useful in a research setting, the findings are not sensitive enough and not specific for HIV disease in a clinical setting. In other words, MRI might miss some HIV-related changes in brain structure or the observed changes might result from other conditions. In general, MRI studies have not been shown to be useful in documenting the degree and progression of HIV-related CNS disease. Moreover, it is still too early after the introduction of potent antiretroviral medications to state conclusively how well HAART has affected the frequency and degree of HIV-related brain abnormalities (other than opportunistic infections of the CNS) detectable by MRI.

MRS may be more sensitive than MRI in detecting neurological changes in HIV/AIDS patients (for a review see Navia and Gonzalez 1997). For example, researchers have measured the levels of a molecule called *N*-acetyl-aspartate (NAA), which serves as an indicator of neuron viability. In those studies, researchers noted changes in NAA levels in brain regions that appeared normal on routine MRI. Several studies found that HIV-infected patients with cognitive impairments had lower NAA levels in white and gray matter than did those without cognitive impairments or HIV-negative control subjects, suggesting neuronal and/or axonal damage in the impaired patients. The NAA changes were largest in white matter of the frontal brain, and preliminary data suggest that the extent of these changes increased with the severity of the patients’ cognitive impairment.

Subsequent MRS studies have measured metabolites called choline-containing compounds (Cho) and myoinositol (mIno), which reflect membrane-related metabolic processes in glia cells. These studies also detected abnormalities in asymptomatic HIV-infected patients whose immune systems were still intact, with increased levels of Cho and mIno primarily in the white matter surrounding the ventricles and in the basal ganglia (see Navia and Gonzalez 1997). This finding is consistent with the neuropathological studies demonstrating glial proliferation in those brain areas. Furthermore, greater increases in Cho and mIno levels were found in patients with more advanced dementia, higher levels of HIV in the cerebrospinal fluid (but not in the blood), and greater reductions in the number of CD4+ cells. Therefore, investigators have suggested that Cho and mIno measures may serve as markers of HIV-induced neuropathological changes or of CNS involvement.

MRS technology also may help clinicians monitor the effects of antiretroviral therapy on the CNS. Changes were observed in repeated MRS studies of patients who received several months of antiretrovial therapy; those patients demonstrated a partial reversal of the metabolic abnormalities (Chang et al. 1999).

### Neuropathology and Neuropsychology of Chronic Alcohol Abusers

Autopsy studies have shown that the brains of chronic alcoholics are smaller, lighter, and exhibit greater atrophy than those of nonalcoholics of the same age and gender (for a review, see Kril and Halliday 1999). This reduced brain size resulted primarily from white matter loss, especially in the frontal lobe. The extent of that white matter loss increased proportionally with the drinker’s maximum daily alcohol consumption. Overall, pathological studies revealed two kinds of alcohol-related damage to the cortex: loss of neurons primarily from the frontal cortex and widespread shrinkage of the neurons (i.e., reduced size of axons and dendrites) throughout the brain. Neuron loss is thought to be permanent whereas the size reduction of neurons and their dendrites is potentially reversible with abstinence. In addition to this cortical damage, severe alcoholics also show reduced size and other abnormalities in various subcortical structures (e.g., the mammillary bodies and thalamus) and in a part of the cerebellum. Most of these findings have been confirmed in animals that were given alcohol in their drinking water over a long period, mimicking lifelong chronic alcohol abuse in humans.

Scientists also had hypothesized that chronic alcohol abusers, who frequently exhibit memory loss and learning difficulties, would show damage to the hippocampus, a brain area associated with memory and learning. However, neuropathological studies detected no significant hippocampal neuron loss in alcoholics; instead, dendrites or metabolism in the hippocampus of alcoholics may be damaged (see Kril and Halliday 1999).

The cerebral cortex is the seat of higher intellectual functions. Consistent with the findings of autopsy studies of chronic alcoholics demonstrating widespread cortical damage, neuropsychological studies frequently have demonstrated cognitive deficits in this population (Oscar-Berman 2000). These include deficits in problem solving, organizing, planning, and abstraction (so-called executive functions that are associated with the frontal lobe); short-term and long-term memory; verbal fluency; learning; and visuospatial perceptions, which generally are associated with subcortical brain structures. Furthermore, the observed cerebellar shrinkage has been associated with impaired coordination and balance commonly found in alcoholics. Taken together, neuropathological and neuropsychological studies suggest that the frontal lobes are particularly susceptible to the deleterious effects of chronic alcohol use in humans but that other structures also are involved.

### Brain Structure and Metabolites in Chronic Alcohol Abusers

Consistent with the neuropathological studies, imaging studies using computed tomography (CT) scans demonstrated atrophy in various brain regions of alcoholics as indicated by shrinkage of the cerebellum and widening of the ventricles and fluid-filled gaps (i.e., sulci) in the brain’s cortex, particularly in the frontal lobe (for reviews see Rosenbloom et al. 1995; Sullivan 2000). MRI studies also demonstrated volume loss in brain regions that show neuron loss in pathological studies, primarily in cortical gray matter regions of the frontal lobes, but also in gray matter of the parietal and temporal lobes. This gray matter loss often is accompanied by increases in fluid volume in the same regions. In addition, MRI studies of chronic alcoholics have demonstrated white matter loss in the frontal-parietal brain and corpus callosum, as well as volume loss of subcortical structures (e.g., the thalamus, hypothalamus, and caudate) and the cerebellum. As did neuropathological studies, neuroimaging studies found no decrease in the size of the hippocampus, despite the memory and learning deficits observed in alcoholics. Researchers have demonstrated some relationships between volume deficits in some brain areas and measures of neuropsychological impairment; however, these relationships have not been consistent.

An alcoholic’s age influences how severe alcohol-related changes in brain structure and function are, with more severe and persistent alcohol-related changes evident in the brains of older alcoholics (Sullivan 2000). Age also appears to affect the brain’s ability to recover from alcohol-related damage. When alcoholics stop drinking, some of the gray and white matter loss and cognitive impairment caused by chronic drinking is reversible. Studies found that the recovery of brain structure and function is greater and more rapid in younger abstinent alcoholics than in older abstinent alcoholics (see Sullivan 2000). The significance of the recovery of brain structures for the recovery of brain functions is under active investigation.

Despite their potential usefulness, MRS studies on the effects of chronic alcohol abuse on the brains of humans or animals are scarce (see Sullivan 2000). Studies of abstinent alcoholics generally suggest lower NAA levels in white matter regions of the frontal and parietal lobes as well as in the cerebellum and thalamus, consistent with neuronal and/or axonal damage in those regions that was not reversible by short-term abstinence. These observations support those of the neuropathological and neuropsychological studies described in the previous section. Patients with alcoholic dementia also reveal evidence of neuronal damage in the basal ganglia and occipital lobe. One MRS study compared certain brain metabolites in the thalamus and frontal cortical regions between alcoholics who had been abstinent for 1 month and alcoholics who had been abstinent for 6 years (see Sullivan 2000). The study found elevated mIno levels after short-term abstinence but not after long-term abstinence, suggesting that some alcohol-induced changes are reversible with prolonged abstinence.

### Comparison of the Brain Regions Affected by HIV Infection and Chronic Alcohol Abuse

Taken together, the pathological, neuropsychological, structural, and metabolic evidence suggests that chronic alcohol abuse predominantly affects the frontal lobe whereas HIV—at least early in the course of the disease—primarily affects white matter deep within the brain as well as subcortical brain structures (see table). Cortical brain regions become involved only during advanced stages of HIV disease, which are associated with cognitive and some clinical impairment. Furthermore, this cortical damage may be delayed by HAART therapy.

Based on these findings, the brain regions expected to be primarily affected in alcohol-abusing HIV-infected subjects include central white matter, subcortical brain structures, and the frontal cortex. These regions are the focus of current structural and metabolic MR studies of the combined effects of alcohol abuse and HIV infection on the brain.

## CNS Effects of Combined HIV Infection and Chronic Alcohol Abuse

The findings reviewed so far strongly suggest that chronic alcohol abuse can adversely affect the natural course of HIV disease. To prove, however, that chronic alcohol abuse exacerbates the CNS effects of HIV infection, researchers must compare alcohol-abusing and nonalcohol-abusing HIV-infected patients on a variety of CNS measures. This section summarizes studies analyzing the interactive effects of HIV infection and chronic alcohol abuse on the brains of patients not treated with HAART.

Using MRS of the brain, Meyerhoff and colleagues (1995) examined both HIV-positive and HIV-negative subjects who were either heavy drinkers (i.e., consumed a mean of 228 drinks per month for at least 3 years prior to study) or light drinkers (i.e., consumed a mean of 14 drinks per month over their lifetime). The light-drinking and heavy-drinking HIV-positive groups were matched for the degree to which their immune systems were already impaired by the HIV infection, and both groups exhibited mild-to-moderate neuropsychological impairment. The study assessed the levels of two molecules called PCr and ATP, which reflect the cell’s energy metabolism, as well as of a group of compounds called PDE, which represent breakdown products of molecules found in the cell membranes.

The study found that chronic alcohol consumption was associated with lower concentrations of PDE, PCr, and ATP in white matter of the region surrounding the ventricles (i.e., the centrum semiovale). Similarly, patients with advanced HIV disease or full-blown AIDS had lower levels of PDE, PCr, and ATP in the white matter compared with both HIV-negative subjects and clinically asymptomatic HIV-positive subjects. The metabolic effects of advanced HIV infection and alcohol abuse were cumulative, because HIV-positive heavy drinkers clearly showed the greatest metabolic deficits in white matter. PDE levels also were significantly lower in subcortical gray matter, which is associated with learning and memory functions, of heavy drinkers with advanced HIV disease compared with HIV-negative light drinkers. These findings are consistent with the neuropathological and neuropsychological observations demonstrating subcortical damage.

Taken together, these findings suggest that both alcohol abuse and HIV infection alter the brain’s energy metabolism and the metabolism of cell membranes and that these alterations are associated with the presence of clinical HIV symptoms. More importantly, the study found that chronic alcohol abuse augmented the adverse effects of HIV on biologically important white matter molecules.

Meyerhoff and colleagues (1997) also conducted a MRS study assessing different molecules, including NAA, in a similar patient population. That study found metabolic changes in the regions of the brain stem—a region at the base of the brain that performs motor, sensory, and reflex functions—of active heavy drinkers. These changes were consistent with pathological evidence for the loss of certain neurons and for gliosis found throughout the brain stem of chronic alcoholics. These changes were particularly pronounced in HIV-positive heavy drinkers, suggesting that HIV infection may have an additional adverse effect on brain stem metabolites.

These studies, which were performed before the introduction of HAART therapy, suggest that chronic alcohol abuse exacerbates some metabolic injury in the HIV-infected brain. Electrophysiological studies, which measure certain aspects of the brain’s electrical activity, have provided additional evidence for an additive effect of heavy drinking on brain damage in HIV-infected subjects. One study assessed an electric signal measurable in the frontal lobe that is thought to reflect mental processes requiring attention and memory updating (Fein et al. 1998). This signal is delayed in HIV-infected subjects, but the delays occurred earlier in the course of HIV disease in alcohol-abusing than in light-drinking or abstinent HIV-infected subjects. Researchers are currently investigating the cognitive and behavioral significance of these brain changes.

The effect of modern antiretroviral treatment (i.e., HAART therapy) on the specific brain alterations observed in alcohol-abusing HIV-positive patients has not yet been investigated thoroughly. Very preliminary data from MRI and MRS studies on light- and heavy-drinking HIV-negative and HIV-positive subjects (with most of the HIV-positive subjects on HAART therapy) detected some of the brain injury commonly associated with HIV infection and with alcohol abuse. The studies found little evidence, however, that chronic alcohol abuse exacerbated the CNS effects of HIV infection in these treated patients.

## Future Outlook

Researchers have begun to investigate the effects of chronic alcohol abuse on HIV-associated CNS disease, integrating studies of immunology, neuropsychology, behavior, and drug-availability (i.e., potential interactions between HIV medications and alcohol) in the body with studies of brain electrophysiology, structure, and function. Longitudinal studies, which follow a group of subjects over extended periods of time, will allow a more powerful assessment of the CNS effects of combined chronic alcohol abuse and HIV disease. Such studies may help answer the question whether aggressive alcoholism treatment can help ameliorate the functional and behavioral consequences of the brain injury associated with HIV disease.

This research also can provide important information on whether and how chronic heavy drinking compromises the effectiveness of emerging antiretroviral therapies, either through behavioral effects on treatment seeking and treatment adherence or through biological effects on the metabolism and levels of antiretroviral medications. Such potential effects on the efficacy of antiretroviral treatments may not only accelerate disease progression in individual patients but, perhaps more importantly, may undermine therapeutic control of the HIV epidemic by allowing drug-resistant HIV strains to emerge in patients who take insufficient doses of their medications.^7^ Current research also will provide important data on the association of heavy alcohol use with continued risk behavior (e.g., unprotected sex or needle sharing) in HIV-infected people, although a discussion of this issue is beyond the scope of this article. If such associations exist, however, they could make chronic heavy drinking in HIV-positive people a particularly potent risk factor for the further spread of HIV.

## Figures and Tables

**Figure:**
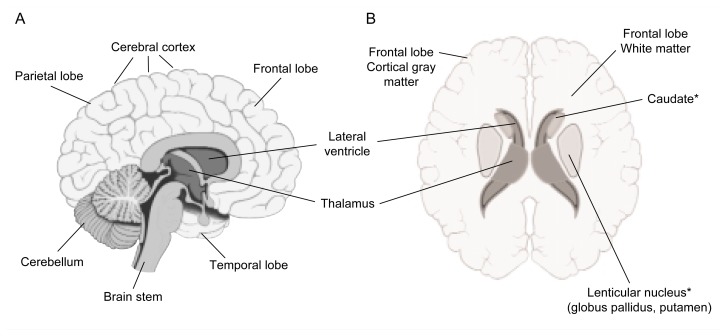
Location of brain areas affected by HIV infection and/or alcohol use. Panel A represents a lengthwise cut through the brain, with the front of the brain facing right. Panel B represents a horizontal (i.e., axial) cut through the brain, with the front of the brain facing up. *Part of the basal ganglia.

**Table:** Brain Regions Affected by HIV Infection and Chronic Alcohol Abuse Based on Pathological, Psychological, Structural, and Metabolic Analyses

Brain Region	Affected by HIV Infection	Affected by Alcohol Abuse
Cortical gray matter	+	++
Frontal lobe	+	++
Parietal lobe	+	++
Temporal lobe	?	+
White matter	++	++
Periventricular	++	++
Supraventricular	++	++
Subcortical	?	++
Subcortical gray matter	++	++
Basal ganglia	++	++
Thalamus	+	+
Hippocampus	?	+
Cerebellum	?	++
Brainstem (midbrain)	?	+

NOTE: ? = no evidence or unknown; + = some evidence; ++ = strong evidence

## Footnotes

1 With the total U.S. population at approximately 270 million, this corresponds to approximately 0.35 percent of the total population.

2 Terms such as “chronic alcohol use,” “alcohol abuse,” “alcoholism,” “heavy drinking,” and similar terms have variable definitions in the literature. Therefore, these terms are used here as in the original studies and reviews cited.

3 For a definition of this and other technical terms used in this article, see the glossary, p. 297.

4 Severe immunosuppression is defined as having less than 200 CD4+ cells per cubic millimeter (mm^3^) of blood; normal levels of CD4+ cells are approximately 1000 cells/mm^3^.

5 These medications derive their names from the fact that they inhibit the functions of two important viral enzymes called reverse transcriptase and protease.

6 Neuropathological studies assess structural brain damage by directly examining brain tissue (e.g., in autopsy studies). Neuropsychological studies evaluate behavioral and cognitive deficits and other psychological symptoms.

7 The experience with tuberculosis offers a precedent for such concerns.

## GLOSSARY

Antiretroviral therapy: Treatment to combat infection with HIV, which belongs to a class of viruses called retrovirus.
Astrocyte: A star-shaped type of *glial cell* that provides structural and functional support to surrounding *neurons* and is important for the regulation of *ion* concentrations.
Axon: A large fiber extending from the cell body of a *neuron* that conducts nerve signals to other *neurons*.
Basal ganglia: Groups of *neurons* located deep within the brain that help control motor and cognitive activity.
CD4+ cell: An immune cell (i.e., lymphocyte) found in the blood that performs important regulatory immune functions, thereby protecting the organism against infections; also called T-helper cell.
Cerebellum: A structure at the base of the brain that is involved in the control of muscle tone, balance, and sensorimotor coordination.
Cerebral cortex: The outer, intricately folded layer of gray matter covering the *cerebrum*; contains areas for processing sensory information, controlling motor functions, speech, higher cognitive functions, emotions, behavior, and memory.
Cerebrospinal fluid: The fluid that surrounds and fills the spaces in the brain and spinal cord.
Cerebrum: The largest portion of the brain; consists of two cerebral hemispheres.
Corpus callosum: A tract of nerve fibers connecting the two hemispheres of the *cerebrum*.
Cytokine: A compound that acts as a chemical messenger between cells in the immune system and other immune and nonimmune cells; if their levels are not controlled tightly, cytokines can damage or kill *neurons*.
Dendrite: A branched projection extending from the body of a *neuron* that relays electric signals from other *neurons* to the cell body.
Glial cells: Brain cells that provide support functions for the *neurons*.
Gliosis: Excessive multiplication of *glial cells*.
Gray matter: Brain tissue consisting mostly of neuronal cell bodies and *dendrites*; constitutes the outer, extensively folded layer of the brain.
Highly active antiretroviral therapy (HAART): Treatment regimen for HIV infection consisting of three medications from different classes of medications that target important viral enzymes; must be taken according to a strict schedule to be most effective.
Ion: An electrically charged atom or group of atoms.
Metabolism: The processes related to the production and breakdown of all types of molecules in the body, whether they serve as structural components of the cells or to provide energy for the body and each cell.
Microglia: A type of *glial cell* that can turn into a type of immune cell called a *phagocyte* or macrophage; can be infected by HIV.
Neuron: A nerve cell; consists of cell body, *dendrites*, and *axon*.
Oligodendrocyte: A type of *glial cell* that forms long extensions containing a protein called myelin; these extensions wrap themselves around *axons* to form a protective sheath.
Opportunistic infection: An infection that can normally be controlled by an intact immune system but that causes illness in a person with a damaged immune system.
Phagocyte: A type of immune cell that seeks out and destroys foreign microorganisms, viruses, and dead cells; also called macrophage.
Subcortical: Located deep inside the brain below the *cerebral cortex*.
Sulci (sulcus): The grooves on the surface of the brain, filled with *cerebrospinal fluid*.
Thalamus: A *gray-matter* structure deep inside the brain that forms the brain’s relay center to the *cerebral cortex*.
Ventricles: Fluid-filled spaces between the brain’s hemispheres or in other organs (e.g., the heart).
White matter: Brain tissue consisting mostly of *glial cells*; *axons*; and myelin, a protective lipid layer surrounding the *axons*.
